# Visual Saliency via Multiscale Analysis in Frequency Domain and Its Applications to Ship Detection in Optical Satellite Images

**DOI:** 10.3389/fnbot.2021.767299

**Published:** 2022-01-13

**Authors:** Ying Yu, Jun Qian, Qinglong Wu

**Affiliations:** School of Information Science and Engineering, Yunnan University, Kunming, China

**Keywords:** visual saliency, selective visual attention, wavelet transform, multiscale saliency map, ship detection

## Abstract

This article proposes a bottom-up visual saliency model that uses the wavelet transform to conduct multiscale analysis and computation in the frequency domain. First, we compute the multiscale magnitude spectra by performing a wavelet transform to decompose the magnitude spectrum of the discrete cosine coefficients of an input image. Next, we obtain multiple saliency maps of different spatial scales through an inverse transformation from the frequency domain to the spatial domain, which utilizes the discrete cosine magnitude spectra after multiscale wavelet decomposition. Then, we employ an evaluation function to automatically select the two best multiscale saliency maps. A final saliency map is generated via an adaptive integration of the two selected multiscale saliency maps. The proposed model is fast, efficient, and can simultaneously detect salient regions or objects of different sizes. It outperforms state-of-the-art bottom-up saliency approaches in the experiments of psychophysical consistency, eye fixation prediction, and saliency detection for natural images. In addition, the proposed model is applied to automatic ship detection in optical satellite images. Ship detection tests on satellite data of visual optical spectrum not only demonstrate our saliency model's effectiveness in detecting small and large salient targets but also verify its robustness against various sea background disturbances.

## Introduction

In the human neural system, a mechanism called selective visual attention has been evolved to facilitate our visual perception to rapidly locate the most important regions in a cluttered scene. Such important regions are said to be perceptually salient because they attract great visual attention. Typically, visual attention is either driven by fast, pre-attentive, bottom-up visual saliency or controlled by slow, task-dependent, top-down cues (Itti et al., 1998; Itti and Koch, 2001; Wolfe and Horowitz, 2004, 2017).

This article is primarily concerned with the automatic detection of bottom-up visual saliency, which has attracted extensive studies by both psychologist and computer vision researchers in the area of robotics, cognitive science, and neuroscience (Borji and Itti, 2013). Just like a bottom-up visual attention mechanism that can rapidly locate salient objects in the human visual pathway, a computational saliency model has the ability to detect the perceptually salient regions in cluttered scenes, which is very useful for object detection, image segmentation, intelligent compression, human fixation prediction, and many more.

One pioneer work concerning the computational modeling of bottom-up visual attention was introduced by Itti et al. (1998) and Itti and Koch (2001). It stimulates the neural mechanism of the human early vision system and has explicit biological rationality. Itti's model (denoted IT) generates a saliency map of the scene under view by modeling the center-surround contrast of intensity, color, and orientation, which is expected to indicate salient regions and predict human fixations. However, since the model is designed conforming to the neuronal architecture of a vision system, it is computationally complex and suffers from over-parameterization. Recently, a kind of algorithm has been designed for salient foreground segmentation. Achanta and Suesstrunk (2010) compute saliency maps by use of the Euclidean distance in the Commission International Eclairage (CIE) LAB space between a given position's value and the maximum symmetric surround mean value of its neighboring area (denoted MSS). Cheng et al. (2015) used a histogram-based contrast (HC) to measure the saliency values of input images. Liu and Yang (2019) exploited color volume and color difference for salient region detection. These algorithms can output fine-resolution saliency maps that highlight large-scale foreground regions. However, since these kinds of algorithms are not biologically motivated, they cannot be used as a model of bottom-up visual attention with psychophysical consistency and often fail to detect salient objects in cluttered scenes.

Another kind of bottom-up saliency model is computed in the frequency domain. These frequency-domain models are not explicitly motivated by a biological mechanism, but they are computationally simple and have good consistency with psychophysics. As a pioneer saliency work of frequency domain, Hou and Zhang (2007) designed a saliency model by use of the Fourier spectral residual (SR) computation. Yu et al. (2009) proposed the pulsed functions of the discrete cosine transform (PCT) to compute visual saliency. Guo and Zhang (2010) introduced a spatiotemporal saliency approach by using a so-called phase spectrum of quaternion Fourier transform (PQFT). Li et al. (2013) compute visual saliency via a scale-space analysis in the hypercomplex Fourier transform (HFT) domain. After that, Yu and Yang (2017) proposed a visual saliency model by using the binary spectrum of Walsh–Hadamard transform (BSWHT).

As for why the frequency domain models can calculate visual saliency, our previous works (Yu et al., 2011a,b) have demonstrated the biological rationality of frequency-domain approaches. These works have verified that whitening or flattening the principal components or the cosine transform coefficients simulates the suppression of the same visual features (iso-feature suppression) in the spatial domain. The iso-feature suppression is just the biological mechanism of bottom-up visual saliency generated in the primary visual cortex (V1) (Zhaoping, 2002; Zhaoping and Peter, 2006). However, due to the excessive suppression of low-frequency components in the image by whitening the principal components, existing frequency-domain models are easy to detect small salient targets, but they have poor ability to highlight large-scale salient regions.

To make the frequency domain model have better detection ability for both large and small salient targets, in this article, we propose a bottom-up visual saliency model based on multiscale analysis and computation in the frequency domain. The proposed model performs multiscale wavelet analysis and computation in the cosine transform domain. It can generate multiscale saliency maps of the scene under view. Unlike the spatial domain approaches, our model computes in the frequency domain, which significantly reduces computational cost for a saliency algorithm. Moreover, the multiscale computation of visual saliency also has biological plausibility because the receptive fields of visual neurons in the primary visual cortex (V1) have various ranges of center-surround mechanism (Itti and Koch, 2001; Zhaoping, 2002; Zhaoping and Peter, 2006). As compared with the existing frequency domain approaches, our model has a better ability to detect small salient objects and meanwhile highlight large-scale salient regions.

Ship target detection in optical satellite images is important in monitoring commercial fishery, oil pollution, vessels traffic, and other marine activities. However, there remain challenges with the ship detection algorithm for its application in a marine surveillance system. One challenge is the existence of sea clutters and heterogeneous regions, which poses difficulties for discriminating ship targets from various background disturbances. Another challenge is that a marine surveillance system needs fast algorithms because it needs to analyze and process large amounts of data in real-time. In this work, we apply our multiscale saliency model to detect the ship signatures in the optical satellite images. It may meet the demands of a marine surveillance system and can detect ships of different sizes accurately. Tests over the Maritime SATellite Imagery (MASATI) dataset prove the robustness and effectiveness of our model when it is applied to ship detection in optical satellite images.

The rest of this article is organized as follows. Section Proposed Model describes the proposed bottom-up visual saliency model based on multiscale analysis and computation in the frequency domain and explains its biological plausibility. Section Experimental Validation presents our model's experiments on psychophysical patterns, eye fixation prediction, and saliency detection for natural images. In section Applications to Ship Detection in Optical Satellite Images, we apply the proposed saliency model to automatic ship detection in optical satellite images. Finally, this article is concluded in section Conclusion and Discussion.

## Proposed Model

This section begins by introducing the proposed model of bottom-up visual saliency step by step, and then gives a complete flow of the model from the input image to a final saliency map.

### Visual Feature Channels

Several works (Treisman and Gelade, 1980; Zhaoping, 2002; Zhaoping and Peter, 2006) have verified that the interaction and integration of the low-level visual features can produce a bottom-up saliency map in the primary visual cortex (V1). To begin with, we will compute these low-level visual feature maps before integrating them as a whole. For a given image ***M*** (e.g., resized to 128 × 128 px), we use ***r***, ***g***, and ***b*** to denote the red, green, and blue color channels of the image, respectively. According to Itti et al.'s (1998) general-tuned color model, one intensity and three general-tuned color feature channels ***I***, ***R***, ***G*, **and ***B*** are calculated as


(1a) $$\begin{array}{r} \text{I}=\frac{\text{r}+\text{g}+\text{b}}{3} \end{array}$$

(1b) $$\begin{array}{r} \text{R}=\text{r}-\frac{\text{g}+\text{b}}{2} \end{array}$$

(1c) $$\begin{array}{r} \text{G}=\text{g}-\frac{\text{r}+\text{b}}{2} \end{array}$$

(1d) $$\begin{array}{r} \text{B}=\text{b}-\frac{\text{r}+\text{g}}{2} \end{array}$$

Note that the general-tuned red, green, and blue channels ***R***, ***G***, and ***B*** are set to zero at locations with a negative value.

In the primary visual cortex (V1) of the human brain, similar neurons have lateral inhibition, that is, excited neurons will inhibit the surrounding similar neurons so that the unique targets in the visual scene are highlighted and become the salient targets obtained by the visual attention mechanism (Zhaoping and Peter, 2006). Referring to the lateral inhibition process, we can consider that a red flower in the green grass is salient. If the color feature energy is considered as the sum of the pixels of the color feature channel, then the green feature channel has the largest energy in the scene. Conforming to the characteristics of selective visual attention, our model adjusts the weight of each color feature channel to reduce the weight factor of the feature channel with large energy. In this article, the weight factors of each feature channel in the visual saliency map are defined as


(2) $$\left\{ \begin{array}{l} \omega_{M}=\frac{\max(M)}{\sqrt{\sum_{i=1}^{128}\sum_{j=1}^{128}M}},\;if\;\sum_{i=1}^{128}\sum_{j=1}^{128}M\;\neq 0\\ \;\omega_{M}=\max(M),\;if\;\sum_{i=1}^{128}\sum_{j=1}^{128}M=0 \end{array}\right.$$

where M denotes any one of the general-tuned feature channels ***I***, ***R***, ***G***, and ***B***, whereas *i* and *j* are the horizontal and vertical coordinates of the corresponding channel.

### Multiscale Saliency Computation in the Frequency Domain

After calculating the visual feature channels of the input image, we perform multiscale saliency computation and analysis in the frequency domain. Given a visual feature channel M, we use the discrete cosine transform (DCT) to transform each visual feature channel of the image into a frequency domain:


(3) $$\begin{array}{r} F=\text{DCT}\left(M\right) \end{array}$$

where “DCT(·)” denotes a 2-dimensional discrete cosine transform, and F is the DCT coefficients matrix of the input visual feature channel. Next, the magnitude matrix ${\text{A}}_{M}$ and the sign matrix ${\text{S}}_{M}$ of the DCT coefficients matrix F are computed as


(4) $$\left\{ \begin{array}{l} A_{M}=\text{abs}(F)\\ S_{M}=\text{sign}(F) \end{array}\right.$$

where the notation “abs(·)” is an absolute value function, and the notation “sign(·)” denotes a signum function. For most input images, the magnitude values of low-frequency coefficients are much greater than those of high-frequency coefficients since the natural images have a strong statistical correlation in the visual space. Our previous works (Yu et al., 2011a,b) have verified that whitening or flattening the principal components or the cosine transform coefficients simulates the suppression of the same visual features (iso-feature suppression) in the spatial domain. The iso-feature suppression is just the biological mechanism of bottom-up visual saliency generated in the primary visual cortex (V1) (Zhaoping, 2002; Zhaoping and Peter, 2006). Most frequency domain-based models (e.g., Yu et al., 2009, 2011a,b; Guo and Zhang, 2010; Yu and Yang, 2017) can detect relatively small salient objects by setting the values of the magnitude matrix to one. For salient objects with very large sizes, they often highlight the contour of a large object because whitening (flattening) the magnitude matrix will lose some important low-frequency information.

To make the frequency domain model have better detection ability for both large and small salient targets, in this work, we propose a bottom-up visual saliency model based on multiscale analysis and computation in the frequency domain. The proposed model not only detect small salient objects but also highlight the whole body of those salient objects with very large size. We consider utilizing the wavelet transform to perform multiscale modulation on the magnitude matrix of the DCT coefficients.

Wavelet transform is widely used in image decomposition and reconstruction, which can decompose an image into multiscale components. In this article, we employ wavelet transform to decompose the magnitude matrix of each visual feature channel and suppress the low-frequency components of the magnitude matrix to a certain extent. Since the salient targets have different sizes in the image, the retention degree of the values in the required magnitude matrix is different. Therefore, we perform multiscale decomposition and reconstruction of the magnitude matrix of each feature channel, and construct a multiscale reconstruction magnitude matrix set $\left\{{\text{A}}_{M,N}^{\prime }\right\}$, where M is the feature channel set, and N denotes the decomposition scale. This process ensures that the optimal reconstructed magnitude matrix of the input image can be retained. In the *j*-scale space, the Mallat decomposition formula of the low-frequency subband is


(5) $$\{\begin{array}{l} B_{\text{ss}}{}_{i,l}^{j-1}=\sum_{k,m}h(k-2i)h(m-2l)B_{\text{ss}}{}_{k,m}^{j}\\ B_{\text{ds}}{}_{i,l}^{j-1}=\sum_{k,m}g(k-2i)h(m-2l)B_{\text{ss}}{}_{k,m}^{j}\\ B_{\text{sd}}{}_{i,l}^{j-1}=\sum_{k,m}h(k-2i)g(m-2l)B_{\text{ss}}{}_{k,m}^{j}\\ B_{\text{dd}}{}_{i,l}^{j-1}=\sum_{k,m}g(k-2i)g(m-2l)B_{\text{ss}}{}_{k,m}^{j} \end{array}$$

and the corresponding reconstruction formula is


(6) $$\begin{array}{l} B_{\text{ss}}{}_{k,m}^{j}\text{​}=\text{​}\sum_{i,l}\left[B_{\text{ss}}{}_{i,l}^{j-1}h(k-2i)h(m-2l)+B_{\text{ds}}{}_{i,l}^{j-1}g(k-2i)h(m-2l\right)\\ \;B_{\text{sd}}{}_{i,l}^{j-1}h(k-2i)g(m-2l)+B_{\text{dd}}{}_{i,l}^{j-1}g(k-2i)g(m-2l)] \end{array}$$

where ***h*** and ***g*** denote low-pass and high-pass filtering, respectively. As has been noted before, the suppression of cosine transform coefficients of an image is equivalent to the suppression of the same visual features (iso-feature suppression) in the spatial domain. Therefore, through such a multiscale decomposition and reconstruction operation upon the magnitude coefficients in the DCT domain, our model simulates the cortical center-surround or iso-feature suppression of various scales in the spatial domain. For this reason, our model can compute the multiscale saliency information simultaneously, which is very helpful to detect salient objects of different sizes.

To recover the multiscale channel conspicuity maps in the visual space, we perform an inverse DCT on the reconstructed magnitude matrix and the corresponding sign matrix ${\text{S}}_{M}$ as


(7) $$\begin{array}{r} {\text{F}}_{M,N}=\text{abs}\left(\text{IDCT}\left({\text{S}}_{M}\cdot {\text{A}}_{M,N}^{\prime }\right)\right) \end{array}$$

where ${\text{F}}_{M,N}$ denotes the N-scale conspicuity map for a given channel M, and “IDCT(·)” is the inverse discrete cosine transform. Afterward, we utilize the obtained one intensity and three-color conspicuity maps at the N-scale to compute the saliency map:


(8) $$S_{N}=\Phi *(\omega_{I}\cdot F_{I,N}+\omega_{R}\cdot F_{R,N}+\omega_{G}\cdot F_{G,N}+\omega_{B}\cdot F_{B,N})$$

where ω_*I*_, ω_*R*_, ω_*G*_, and ω_*B*_ denote the weight factors of corresponding feature channels ***I***, ***R***, ***G*, **and ***B***, which are calculated by using Equation (2). The notation Φ denotes a 2-dimensional Gaussian low-pass filter. The notation ${\text{S}}_{N}$ is the N-scale saliency map of the input image.

### Final Saliency Map

To generate the optimal visual saliency map from the multiscale saliency maps $\left\{{\text{S}}_{N}\right\}$, we introduce an evaluation function to evaluate the multiscale saliency maps. More often than not, the more complete the salient region in multiple saliency maps of the same scene, the better the saliency map with less background interference. The evaluation function is defined as the noise coefficient of the saliency map multiplied by the information entropy, where the noise coefficient is the sum of the product of the pixels corresponding to the background interference matrix and the saliency map matrix. According to the visual attention characteristics that the central area of the image is more likely to become the salient region, the background interference matrix is constructed as a gradient matrix with the same resolution as the saliency map, with a maximum value of 1 and a minimum value of 0. Information entropy is often used as the quantitative standard for evaluating images. In this work, we use information entropy to characterize the degree of confusion of a saliency map. The greater the entropy, the more chaotic the saliency map, that is, the more background interference. Therefore, for a given saliency map *S*, the corresponding evaluation function *H* can be defined as


(9) $$\begin{array}{r} H=E\underset{x}{\sum }\underset{y}{\sum }\text{S}\left(x,y\right)\text{K}\left(x,y\right) \end{array}$$

where *E* is the information entropy of the saliency map **S**, and **K** denotes the background interference matrix. *x, y* are the horizontal and vertical coordinates of a matrix. According to the definition of the evaluation function, the smaller the function value, the better the saliency map.

To improve the adaptability of the model in this article, two saliency maps with the lowest value of the evaluation function are selected. Next, the evaluation function values of the corresponding saliency map are exchanged as coefficients to construct the fusion map, and the fusion map is used as the final saliency map ***§*** after central bias optimization. This calculation process is formulated as


(10) $$§=\psi \cdot (H_{2}S_{1}+H_{1}S_{2})$$

where **ψ** is the central bias matrix. **S**_1_ denotes the saliency map with the smallest evaluation function value, whereas **H**_1_ is the corresponding evaluation function value of **S**_1_. When there is little difference in the values of the evaluation function, the two saliency maps generate the final saliency map close to their mean value. When the difference of *H*_1_ and *H*_2_ is large, **S**_2_ has a weak effect on the generation of the final saliency map.

To sum up, the proposed computational model from input image ***M*** to final saliency map ***§*** is as follows:

Step 1. Compute one intensity and three general-tuned color feature channels ***I***, ***R***, ***G*, **and ***B*** by using Equation (1), and calculate the weight factor $\omega_{M}$ for each feature channel by using Equation (2).

Step 2. Perform a DCT transformation on each feature channel, and calculate the magnitude matrix ${\text{A}}_{M}$ and the sign matrix ${\text{S}}_{M}$ of the DCT coefficients by using Equations (3) and (4).

Step 3. Perform multiscale wavelet transform on all feature channels to obtain the multiscale reconstruction magnitude matrix set $\left\{{\text{A}}_{M,N}^{\prime }\right\}$ by using Equations (5) and (6).

Step 4. Performing an inverse DCT transformation on the magnitude matrix ${\text{A}}_{M,N}^{\prime }$ and the corresponding sign matrix ${\text{S}}_{M}$ to compute the N-scale conspicuity map ${\text{F}}_{M,N}$ by using Equation (7).

Step 5. Performing a weighted summation of the conspicuity map of all four feature channels to compute the N-scale saliency map $S_{N}$ by using Equation (8).

Step 6. Compute the evaluation function value of the N-scale saliency map ${\text{S}}_{N}$ by using Equation (9).

Step 7. The two saliency maps with the smallest value of the evaluation function are selected to generate a final saliency map ***§*** by using Equation (10).

**Figure 1:**
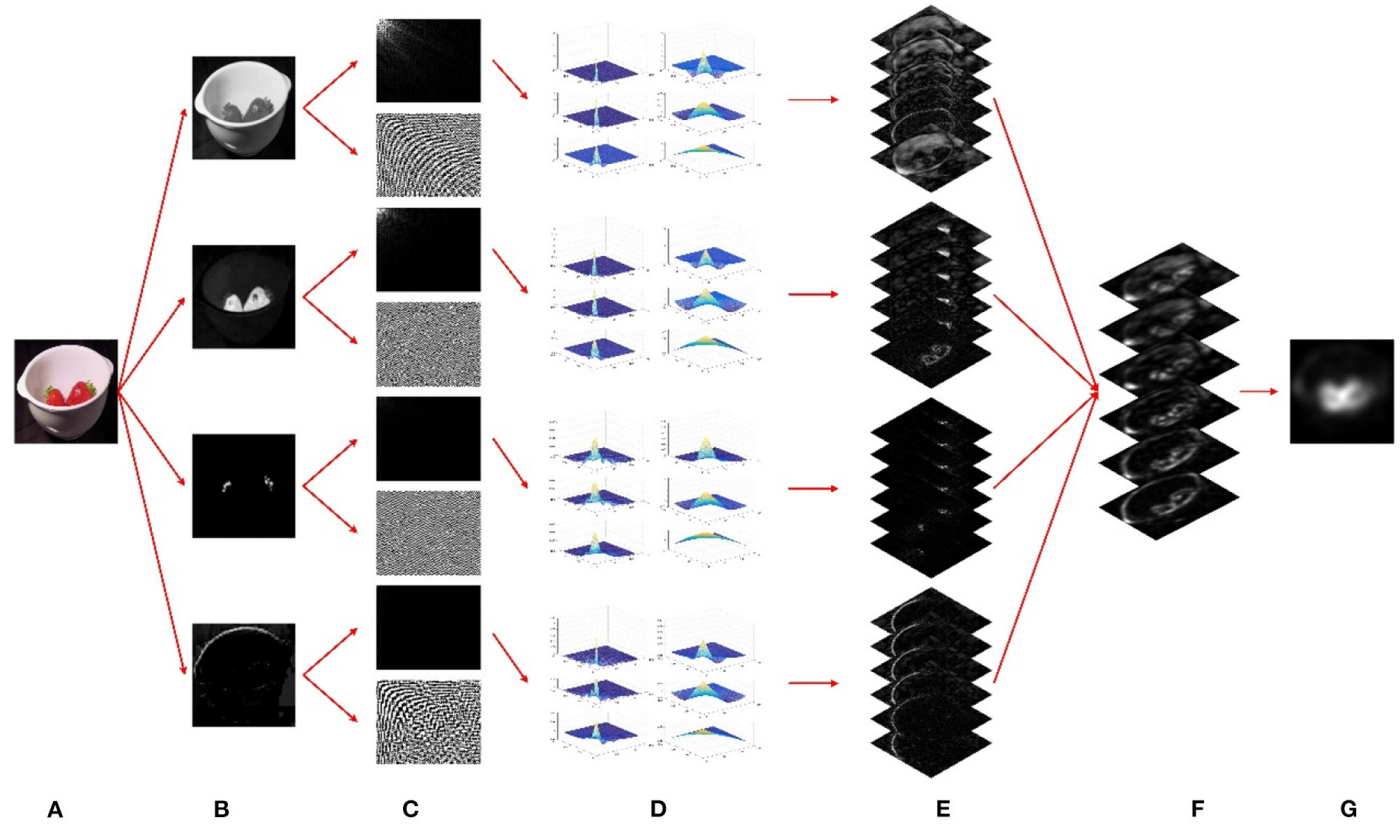
An illustration of our model for saliency detection. **(A)** Input image. **(B)** Four general-tuned visual channels. **(C)** Discrete cosine transform (DCT) magnitude spectra and corresponding signum spectra. **(D)** Wavelet decomposed multiscale magnitude spectra for every single channel. **(E)** Six multiscale conspicuity maps for every single channel. **(F)** Six multiscale saliency maps after channel-wise integration. **(G)** A final saliency map after scale-wise combination.

The complete flow of the proposed model is illustrated in Figure 1. We initially resize the input image to a suitable scale and decompose it into the general-tuned intensity, red, green, and blue feature channels. Each of the four general-tuned feature channels is subjected to a DCT. Next, we use a multiscale wavelet transform to decompose the DCT magnitude spectrum of each channel and then obtain the decomposed multiscale magnitude spectra for every single channel. Afterward, the decomposed magnitude coefficients are subjected to an inverse DCT so that the six multiscale conspicuity maps of each feature channel can be generated. Then, for each scale, we integrate the four conspicuity maps to form a saliency map. Finally, a final saliency map is obtained by combining the two multiscale saliency maps with the smallest *H*-value. Note that the saliency map is a topographically arranged map that represents the visual saliency of a corresponding visual scene. It can be seen from Figure 1 that the salient objects are the strawberries, which pop out from the background in the final saliency map.

It is worth noting again that the flattening modulation of image frequency domain coefficients approximately simulates the suppression of the same visual features (iso-feature suppression) in the spatial domain. Such a mechanism of iso-feature suppression generates bottom-up visual saliency in the primary visual cortex (V1). In this work, we employ multiscale frequency domain modulation by using a multiscale wavelet transform on the magnitude coefficients in the DCT domain. This calculation process is equivalent to flattening the frequency domain coefficients in different degrees (see Figure 1D), rather than in a single way, to calculate the multiscale visual saliency (see Figure 1F) in the spatial domain.

## Experimental Validation

In this section, we compare our model with eight bottom-up saliency approaches: Itti's model (IT) (Itti et al., 1998), maximum symmetric surround mean value (MSS) (Achanta and Suesstrunk, 2010), histogram-based contrast (HC) (Cheng et al., 2015), spectral residual (SR) (Hou and Zhang, 2007), pulsed cosine transform (PCT) (Yu et al., 2009), PQFT (Guo and Zhang, 2010), hypercomplex Fourier transform (HFT) (Li et al., 2013), and binary spectrum of Walsh-Hadamard transform (BSWHT) (Yu and Yang, 2017). All saliency approaches are conducted on psychophysical pattern tests, human eye fixation prediction, and saliency detection for natural images. The experiments provide an objective evaluation as well as a visual comparison of all saliency maps. Moreover, we give a comparison of the computational time cost of all saliency approaches.

In the experiments of human eye fixation prediction and natural image saliency detection. We will employ three popular objective evaluation metrics: the precision-recall (P-R) curve (Davis and Goadrich, 2006), the receiver operating characteristic (ROC) curve (Tatler et al., 2005), and the area under the curve (AUC). For each saliency map, several binary maps are generated by segmenting the saliency map with a threshold τ varying from 0 to 255. We can obtain the true positive (*TP*), the false positive (*FP*), the false negative (*FN*), and the true negative (*TN*) by comparing a binary map with the ground truth (GT) map. Then, the *Recall* and the *Precision* metrics for a binary map can be calculated as


(11) $$\left\{ \begin{array}{l} Recall=\frac{TP}{TP+FN}\\ Precision=\frac{TP}{TP+FP} \end{array}\right.$$

The P-R curve can be plotted with the averaged *Precision* vs. *Recall* values overall saliency maps generated from a saliency approach. Moreover, we compute the true positive rate (*TPR*) and the false positive rate (*FPR*) according to the following formulas:


(12) $$\left\{ \begin{array}{l} TPR=\frac{TP}{TP+FN}\\ FPR=\frac{FP}{FP+TN} \end{array}\right.$$

The ROC curve can be plotted with the averaged *TPR* vs. *FPR* values overall saliency maps generated from a saliency approach. Then we compute the area under the ROC curve that is denoted as a ROC-AUC score. Note that most published articles use these three metrics to evaluate a saliency map's ability to predict eye fixations or detect salient regions.

### Psychophysical Consistency

**Figure 2:**
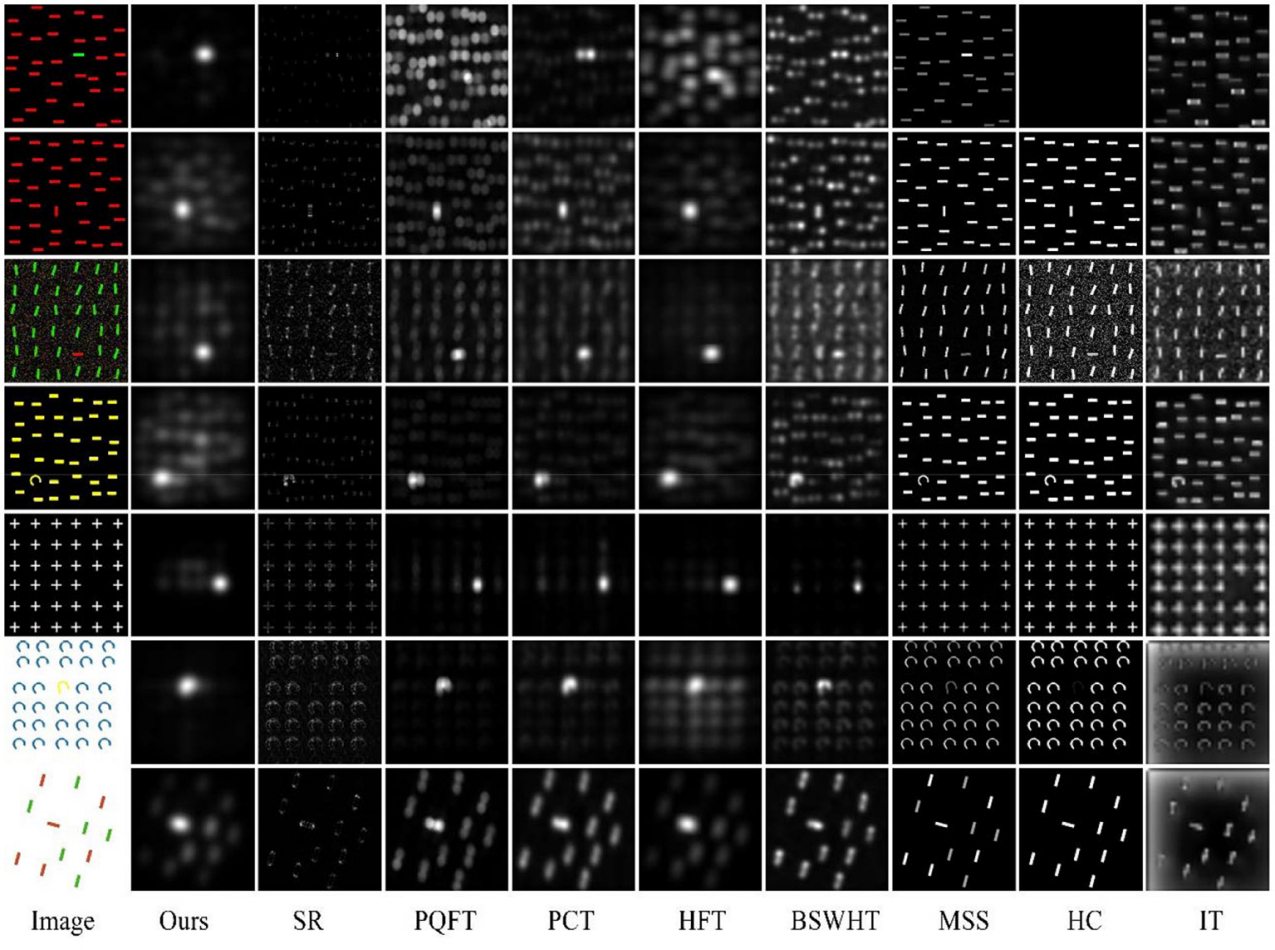
Test on psychophysical patterns for all 9 saliency approaches.

Psychophysical patterns have been widely used in attention selection tests not only to explore the mechanism of bottom-up attention but also to evaluate the saliency models (e.g., Itti et al., 1998; Hou and Zhang, 2007; Yu et al., 2009, 2011a,b; Guo and Zhang, 2010; Li et al., 2013). Figure 2 shows the saliency maps of all saliency approaches on seven psychophysical patterns (including salient targets of unique color, orientation, shape, missing feature, or conjunction feature). It can be seen that IT, MSS, and HC fail to detect (highlight) the salient targets with distinctive orientation or shape. SR cannot detect color saliency since it only computes in an intensity channel. As frequency-domain approaches, PQFT, PCT, HFT, BSWHT, and our method (denoted as “Ours”) can successfully detect salient objects with distinctive orientation or missing features (the 5th pattern). It can be noticed that PCT and our method can find all salient objects with distinctive colors; whereas PQFT and HFT cannot highlight the color pop-out in the 1st pattern. In this test, PCT and our method are the best performers, which are highly consistent with human perception in these psychophysical patterns.

It is worth stating that this article proposes a visual saliency method based on frequency domain calculation. At present, all frequency-domain visual saliency methods do not calculate pixel by pixel, and the output saliency map does not have a clear and accurate object contour. Before outputting the final saliency map, these frequency-domain methods need to do low-pass filtering to obtain an applicable and smooth visual saliency map. Nevertheless, the advantages of frequency-domain methods are also obvious. They can indicate the salient positions and regions in the visual scene, and can better predict the gaze or fixations driven by a human's bottom-up attention mechanism.

### Eye Fixation Prediction

In this subsection, we validate the proposed saliency maps by use of the dataset of 120 color images from an urban environment and corresponding human eye fixation data from 20 subjects provided by Bruce and Tsotsos (2009). These images consist of indoor and outdoor scenes, of which some have very salient items, and others have no particular regions of salience.

To quantify the consistency of a particular saliency map with a set of fixations of the image, wey employ the ROC-AUC score as an objective evaluation metric. It is worth noting that the ROC-AUC score is sensitive to the number of fixations that are used in the calculation. Former fixations are more likely to be driven by the bottom-up manner, whereas later fixations are more likely to be influenced by top-down cues. In this test, we calculate the ROC-AUC scores for each image by using all fixations and repeating the process but using only the first two fixation points. Table 1 lists the ROC-AUC score averaged over all 120 images for each saliency approach. As can be seen, our method obtains the highest ROC-AUC scores in both tests and therefore has the best capability for predicting eye fixations.

**Table 1:** The receiver operating characteristic (ROC)- area under the curve (AUC) scores of all nine saliency methods.

**Method**	**Ours**	**SR**	**PQFT**	**PCT**	**HFT**	**BSWHT**	**MSS**	**HC**	**IT**
All fixations	0.7889	0.6228	0.7570	0.7605	0.7653	0.7761	0.6558	0.5766	0.5365
First fixations	0.8252	0.6274	0.7696	0.7723	0.7902	0.7913	0.6698	0.5850	0.5444

**Figure 3:**
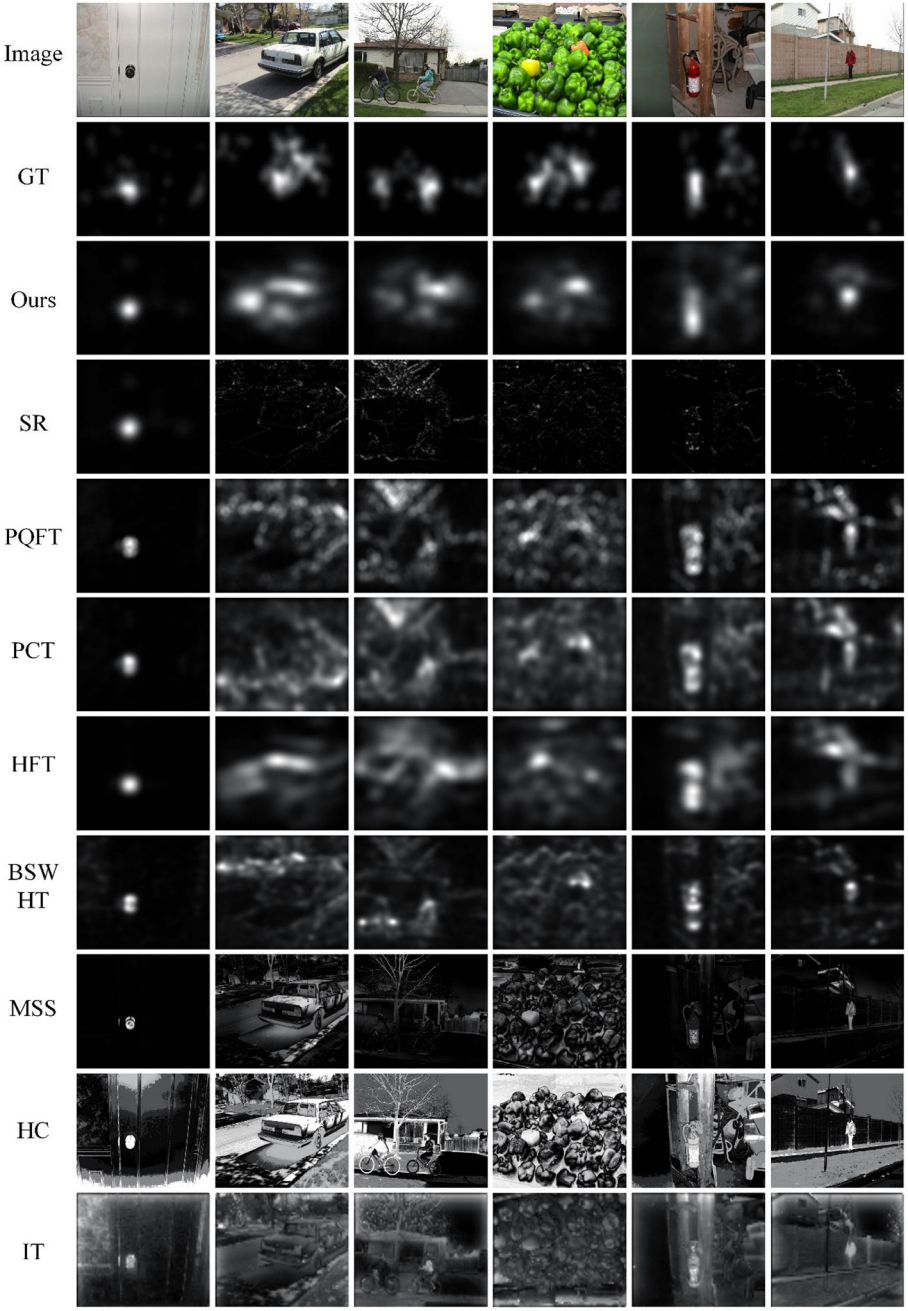
Qualitative analysis of the saliency maps for eye fixation prediction.

Figure 3 gives the saliency maps for six representative images from the data set, which provides a qualitative comparison of all saliency methods. We generate corresponding ground truth images by using a Gaussian filter to perform convolution on the fixation map for all subjects. Some of these images have small salient objects, and others have large-scale regions of interest. Analyzing the qualitative results, we can see that our method shows more resemblance to the ground truth than the other 8 saliency approaches. The regions highlighted by our proposed method overlap to a surprisingly large extent with those image regions looked at by humans in free viewing. Good performance concerning color pop-out is also observed with our method as compared to other approaches. MSS, HC, and IT can obtain fine resolution saliency maps, but they are more likely to focus on large-scale structures and thereby miss some small salient objects.

### Saliency Detection for Natural Images

**Figure 4:**
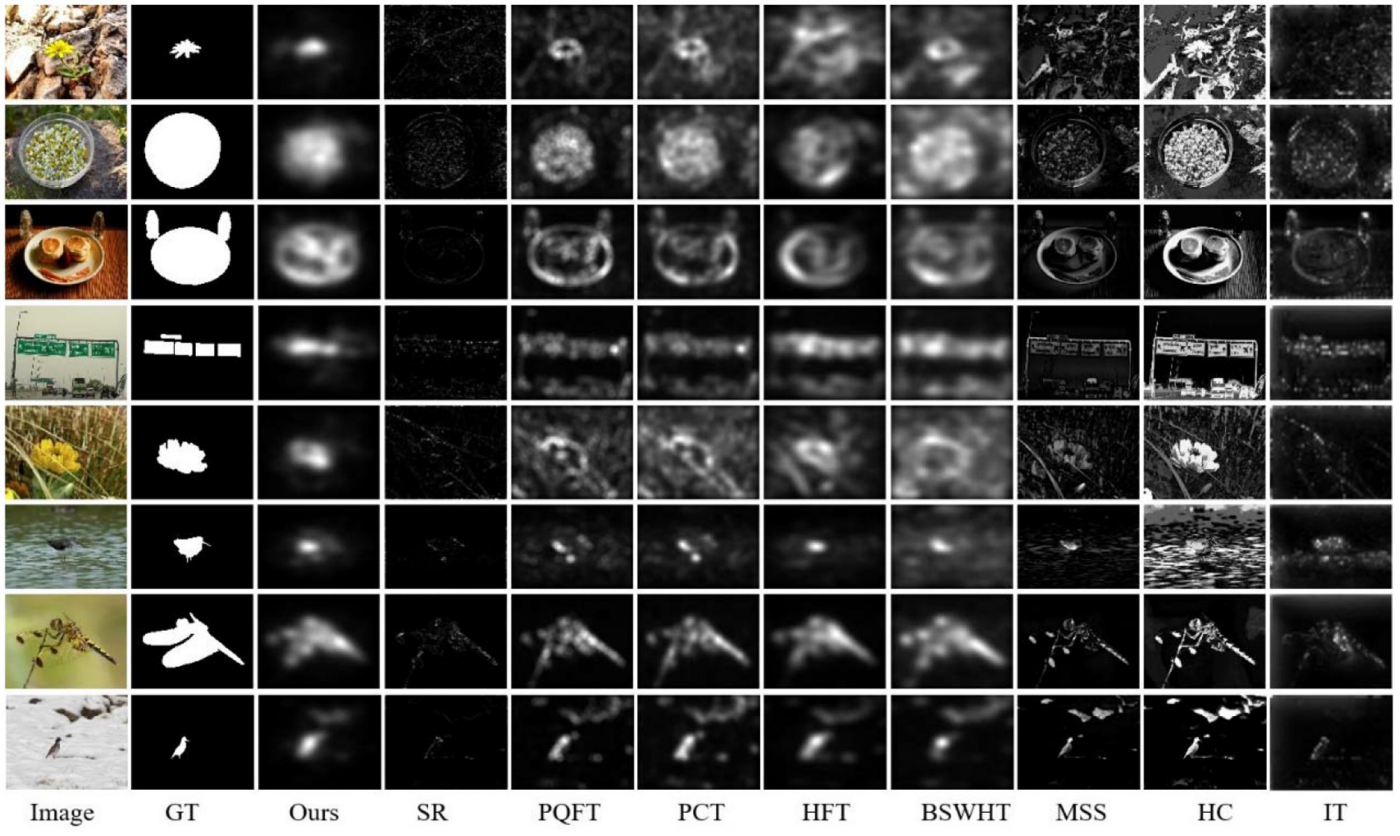
Visual comparison of all saliency approaches on ECSSD dataset.

In this subsection, we compare our method with 8 other saliency approaches on the Extended Complex Scene Saliency Dataset (ECSSD) dataset (Shi et al., 2016) that includes 1,000 natural images and corresponding GT images. Figure 4 gives the saliency maps for eight sample images from the ECSSD dataset, which provide a visual comparison of all saliency methods. It can be seen that MSS, HC, and IT can obtain high-resolution saliency maps, but they suffer from cluttered backgrounds. PQFT, PCT, HFT, and BSWHT can detect small salient objects effectively, but sometimes they fail to highlight the whole salient objects with relatively large size. Note that our proposed method can enhance the salient regions and meanwhile suppress background clutters heavily. Moreover, since our method computes visual salience in a multiscale manner, it can detect both small and large scale salient regions simultaneously.

**Figure 5:**
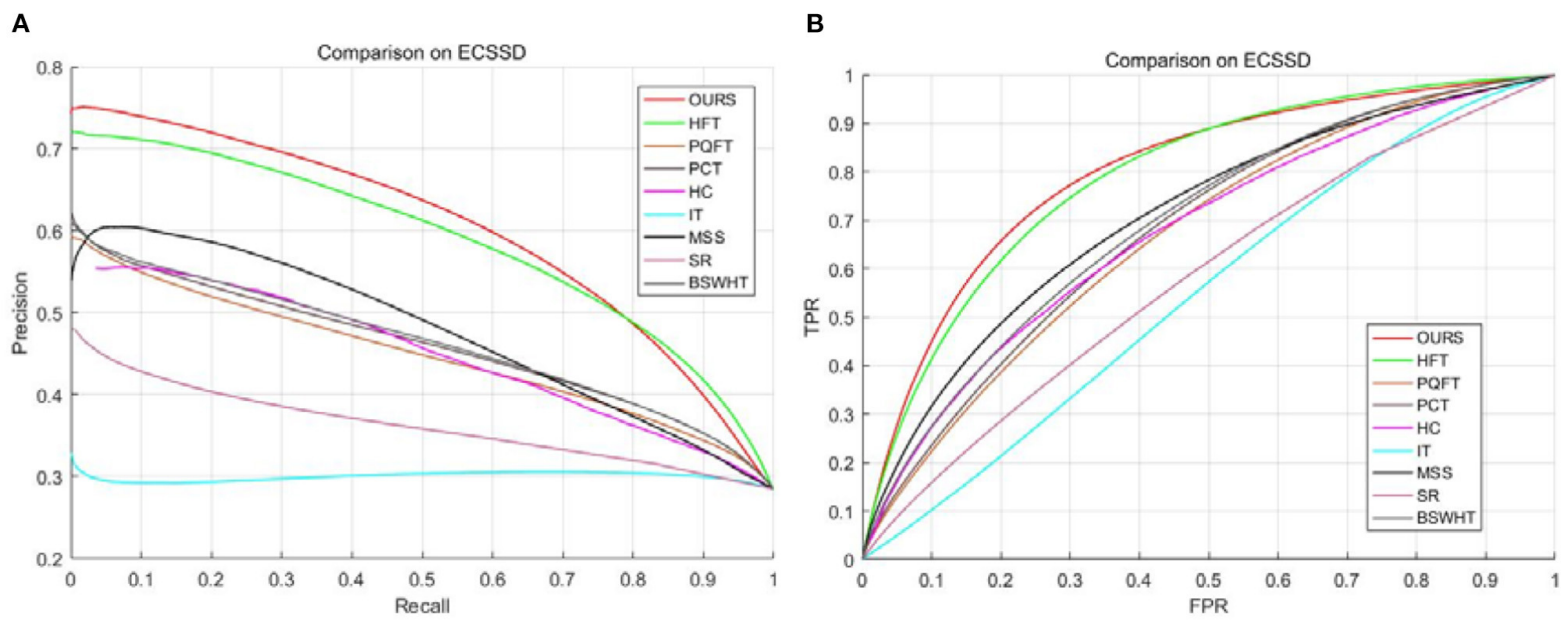
**(A)** P-R curves on the ECSSD dataset. **(B)** ROC curves on the ECSSD dataset.

To evaluate the detection accuracy objectively, we plot the P-R curves and the ROC curves for all saliency approaches as shown in Figures 5A,B. Note that a high ROC or P-R curve indicates the saliency maps have a high resemblance with the GT images. As can be seen, our method and HFT obtain comparatively high curves as compared to other saliency approaches. Nevertheless, it can be noticed that our method is slightly better than HFT. Table 2 lists the ROC-AUC score averaged over all 1,000 images for each saliency method. As expected, our method obtains the highest ROC-AUC score. This means that our method achieves the best performance in this saliency detection test.

**Table 2:** The ROC-AUC scores of all nine saliency methods.

**Method**	**Ours**	**SR**	**PQFT**	**PCT**	**HFT**	**BSWHT**	**DN**	**MSS**	**HC**	**IT**
AUC	0.7990	0.5805	0.6681	0.6813	0.7895	0.6954	0.6333	0.7091	0.6755	0.5493

It should be noted that this article mainly studies the computation of bottom-up visual saliency. Bottom-up attention or saliency studies mostly use psychophysical patterns (section Psychophysical Consistency) and Bruce and Tsotsos's eye fixation prediction dataset (section Eye Fixation Prediction). These two datasets were created specifically for the bottom-up attention tests. For more testing, we conducted tests on the ECSSD dataset in this section. The ECSSD dataset is particularly used for foreground region segmentation methods. They are not only purely bottom-up but also need more top-down calculations. Nevertheless, our method still achieves good performance in this test.

### Computational Time Cost

Computational speed is an important metric to evaluate the performance of a saliency model. We also record the computational time cost per image from the ECSSD dataset in a standard desktop computer environment. Table 3 gives each method's Matlab runtime measurements averaged over the data set. It can be seen that the traditional frequency-domain models (SR, PQFT, PCT, and BSWHT) are relatively faster than other methods. As a multiscale frequency domain calculation model of visual saliency, our method needs about 10 times the computational cost of the traditional frequency-domain model. Nevertheless, it has about the same computational cost as HFT and MSS and is still faster than HC and IT. Note that all saliency methods are implemented on such a computer platform as Intel i7-8650U 1.90GHz CPU, and 16GB of memory.

**Table 3:** Computational time cost per image for all saliency methods over the ECSSD dataset.

**Method**	**Ours**	**SR**	**PQFT**	**PCT**	**HFT**	**BSWHT**	**MSS**	**HC**	**IT**
Time(s)	0.1272	0.0109	0.0163	0.0147	0.1183	0.0102	0.0934	0.3349	0.2224

## Applications to Ship Detection in Optical Satellite Images

In this section, we apply the proposed method to detect ship signatures in optical satellite images. To validate the effectiveness of our method, we conduct experiments by use of real optical satellite images from the MASATI dataset (Antonio-Javier et al., 2018). All tests in this section are run on a Windows platform (Microsoft Incorporation, US). The computer is equipped with a quad-core Intel 2.9 GHz CPU and 32 GB of memory (Intel Incorporation, US). All the program codes are implemented in the MATLAB (MathWorks Incorporation, US) R2017b environment.

### Saliency-Based Ship Detection in Optical Satellite Images

Automatic ship detection in optical satellite images has attracted intensive investigations (Bi et al., 2012; Jubelin and Khenchaf, 2014; Qi et al., 2015; Zou and Shi, 2016; Li et al., 2020). It plays a crucial role in a maritime surveillance system. Some studies perform ship detection by using synthetic aperture radar (SAR) (Crisp, 2004; Yu et al., 2011a). However, strong speckles (caused by the coherence of backscattered signals) pose great difficulties for an automatic ship detection system. Compared with the SAR data, optical satellite images can provide more detailed characteristics of ship signatures.

More often than not, automatic ship detection will encounter two challenges. First, a marine surveillance system needs fast algorithms since it has to deal with a large amount of data in real-time. Second, lots of background disturbances always exist in the optical satellite images. Conventional target detectors use a constant false alarm rate (CFAR) which automatically adapts to the statistical distribution of sea clutters and targets of interest (Chen and Reed, 1987; Reed and Yu, 1990; Yu and Reed, 1993). However, if the signature of a target has similar intensities as its surroundings, the CFAR detector cannot discriminate the targets from their background clutters. It should be noticed that human vision is superior to existing techniques in observing a slick in the surrounding sea, and some vessels undetected by conventional algorithms are visible to the eye. Motivated by this fact, we employ our proposed saliency method to perform ship detection in optical satellite images.

Since ships are visually salient and will become dominant locations in a saliency map, a constant threshold value can be employed to discriminate the ship targets from sea backgrounds. However, a constant threshold will produce false alarms when no ship target appears in the scene under view. Therefore, we consider designing an adaptive threshold to detect the ship targets. The threshold value is computed by using the saliency values of the given saliency map:


(13) $$\begin{array}{r} T_{s}=\alpha \left(\mu_{s}+2\sigma_{s}\right) \end{array}$$

where μ_*s*_ and σ_*s*_ are, respectively, the mean value and the standard deviation of the final saliency map, and α is an empirically tuned parameter. Note that a small α may lead to false alarms although it can detect ship targets; whereas a large α is likely to miss some ship signatures although it avoids false alarms. Through lots of experiments, we find that the detection results are reasonable when the parameter α = 4.

An important note about our method's application to ship detection in optical satellite images is that the saliency map should be computed at full resolution. This is different from the salience computation for a natural image. Note that great disparities may exist in the size of various ships, and our method can detect both small and large salient objects simultaneously when the saliency map is computed at a high resolution. Therefore, to obtain high-resolution saliency maps with well-defined boundaries of targets, we directly use full-resolution optical satellite images to compute their saliency maps. This computation process can be considered as a human looking at the scenes at a fine resolution in a very careful manner.

### Test on the MASATI Dataset

**Figure 6:**
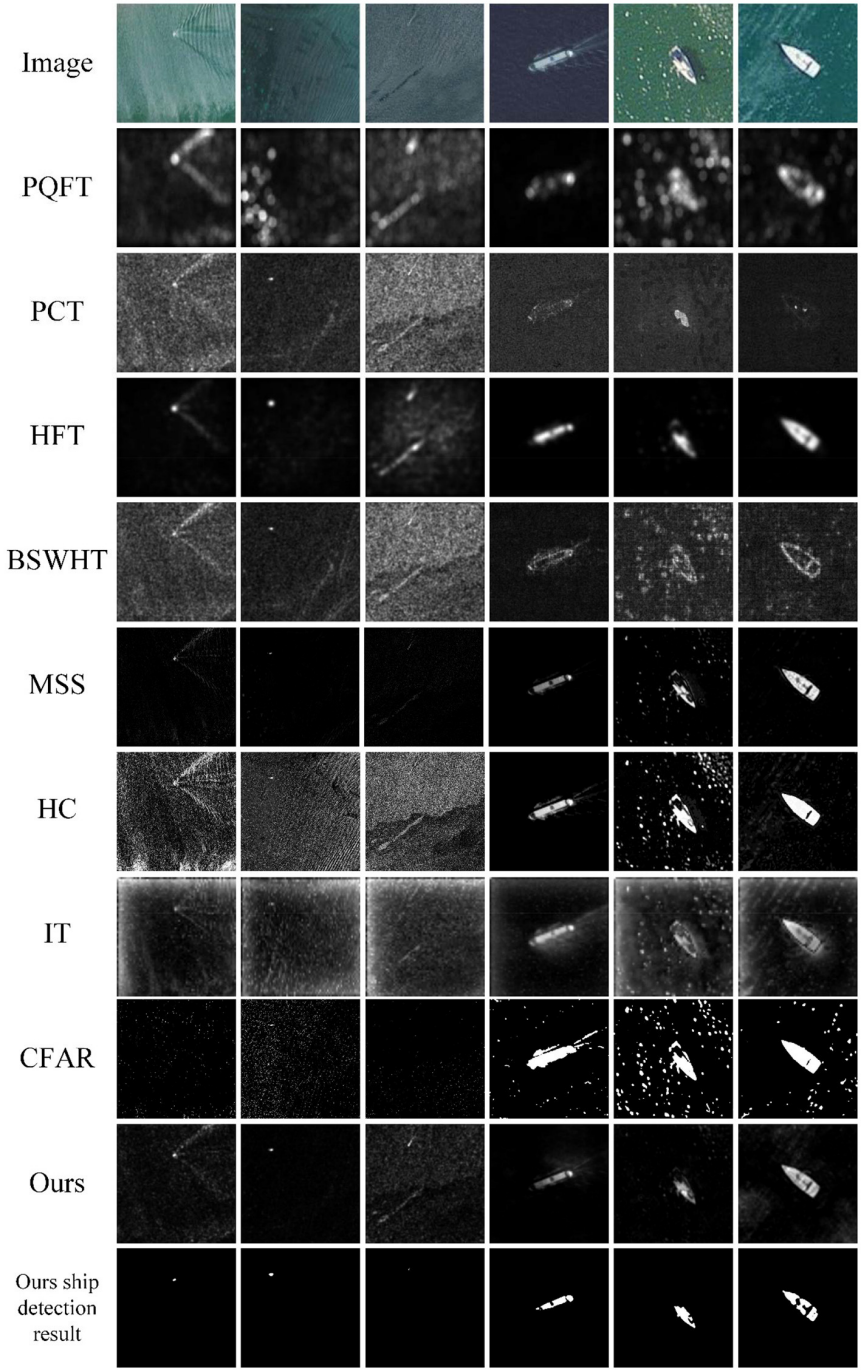
A visual comparison of the saliency maps obtained by all eight approaches, as well as the detection results of CFAR and our method for the MASATI images with a single ship target.

**Figure 7:**
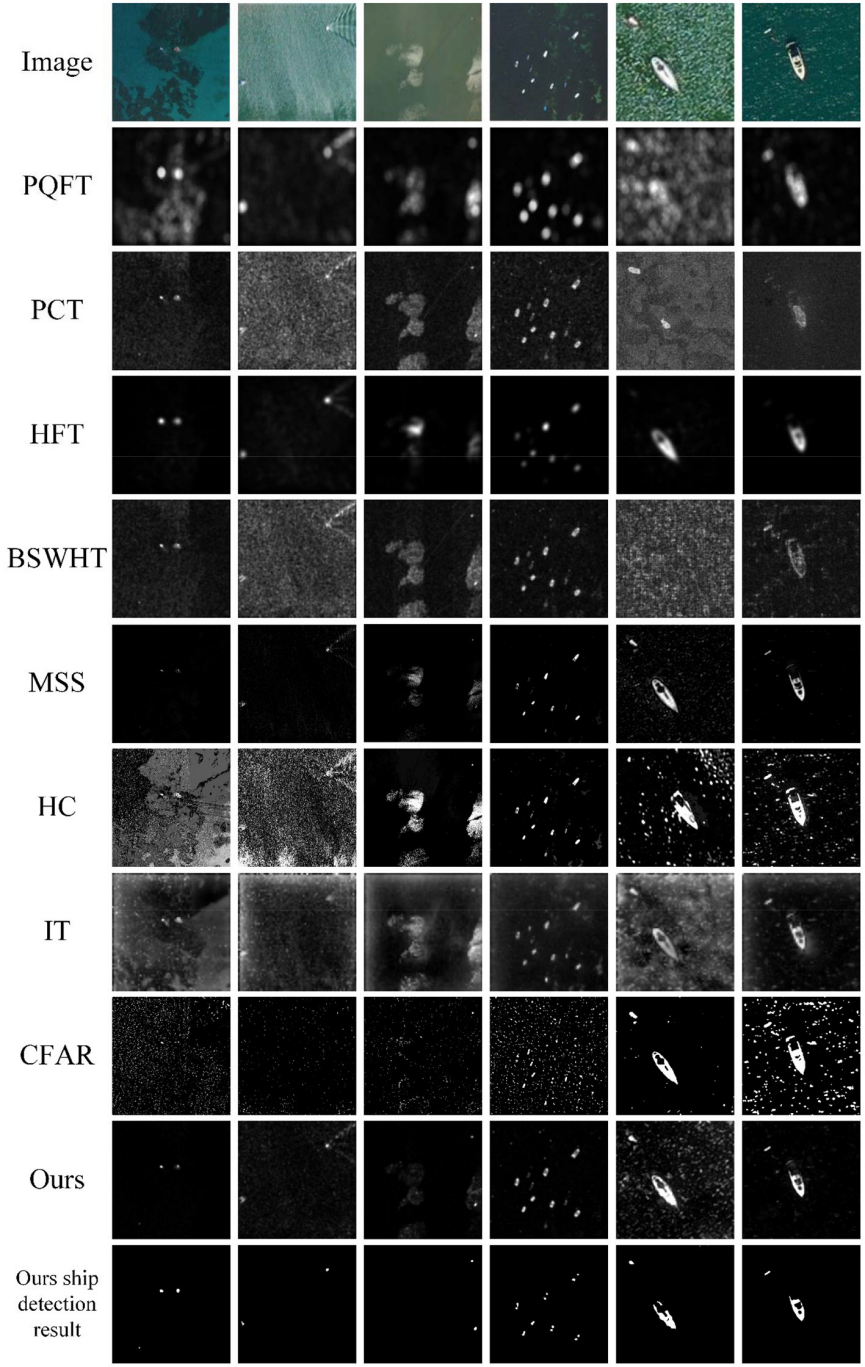
A visual comparison of the saliency maps obtained by all eight approaches, as well as the detection results of CFAR and our method for the MASATI images with multiple ships.

We conduct our method over the MASATI dataset that contains 6,212 satellite images in the visible spectrum. The dataset was collected from Microsoft Bing Maps (Antonio-Javier et al., 2018), of which each image has been manually labeled according to various classes. Since our tests only concern ship detection from sea backgrounds, we choose three sub-classes: *ship, multi*, and *detail* to test our multiscale saliency-based ship detection method. The *ship* sub-class represents images where a single ship appears within the image. The *multi* sub-class describes other images in which two or more instances of ships appear within them. In both sub-classes, the ships have lengths between 4 and 10 pixels. The *detail* sub-class are images with large-scale ships within a length between 20 and 100 pixels. The images were captured in RGB, and the average image size has a spatial resolution of around 512 × 512 pixels. The dataset was compiled between March and September of 2016 from different regions in Europe, Africa, Asia, the Mediterranean Sea, and the Atlantic and Pacific Oceans. We cannot provide simultaneous ground truths at present; nevertheless, the referred targets can be visually interpreted from these optical satellite images. Some typical test results are shown in Figures 6, 7.

Figure 6 shows six sample images with a single ship target from the *ship* and the *detail* sub-classes of the MASATI dataset. The images contain disturbances of ship wakes, sea waves, clutters, and heterogeneities, which will cause challenges for a ship detection task. The 2nd−10th rows of Figure 6 present the saliency maps of 7 comparison saliency approaches, the detection results of CFAR, and the saliency maps and detection results of our method, respectively. It can be seen that PQFT, PCT, BSWHT, HC, and IT cannot suppress the background disturbances effectively, particularly for the images with small ships. Although PQFT, BSWHT, MSS, HC, and IT can detect large ships, they fail to uniformly highlight the whole salient regions for these large-scale targets. It seems that HFT finds both small and large targets in this test, but it highlights some heterogeneous regions in the 3rd image. The CFAR method fails to detect small ship targets whereas it causes false alarms even though it works at a low false alarm rate. It should be noted that both small and large ship locations in our saliency maps can pop out relative to the clutter backgrounds and therefore are successfully detected by our method.

Figure 7 shows six sample images with multiple ship targets from the *multi* and the *detail* sub-classes of the MASATI dataset. This test is somewhat difficult because the sample images comprise strong disturbances including reefs, ship wakes, cloudlets, heterogeneities and clutters of seawater, etc. Moreover, there may exist a huge disparity in the size of the ships in a scene (5th and 6th images). The 2nd−10th rows of Figure 7 present the saliency maps of 7 comparison approaches, the detection results of CFAR, the saliency maps, and the detection results of our method, respectively. Since PQFT, PCT, and BSWHT only compute visual saliency on a single scale, they cannot effectively suppress the background disturbances for these cluttered scenes. Note that HC, MSS, and IT compute visual salience in the spatial domain. They cannot suppress the cloudlets or other disturbances effectively. The CFAR detector inherently has numerous false alarms and cannot discriminate ships from these false alarms. It can be seen that our method highlights the ships and meanwhile suppresses the background disturbances in the saliency maps. Since our method can compute multiscale visual saliency, it accurately finds both small and large ship targets in this difficult test.

## Conclusion and Discussion

This article investigates automatic detection of bottom-up visual saliency from the perspective of multiscale analysis and computation in the frequency domain. We manifested that multiscale saliency information can be computed by performing multiscale wavelet decomposition and computation upon the magnitude coefficients in the frequency domain. The proposed model simulates the multiscale cortical center-surround suppression and has biological plausibility. The model is fast and can provide multiscale saliency maps, which are important for detecting salient objects of different sizes. Experiments over psychophysical patterns and natural image datasets showed that the proposed model outperforms state-of-the-art saliency approaches when evaluated by the ability to predict human fixations, and by the objective metrics of the P-R curves and the ROC-AUC scores. The applications to ship detection in optical satellite images proved that the proposed multiscale visual saliency model is very effective in detecting both small and large ship targets simultaneously from the surrounding sea and robust against various background disturbances.

The main contribution of this article is to extend the traditional frequency-domain visual saliency model to multiscale saliency calculation. The traditional visual saliency model uses single-scale frequency domain calculation, while our new model uses multiscale frequency domain calculation. The multiscale visual saliency calculation is realized by decomposing the frequency domain coefficients of the input image by multiscale wavelet transform. The traditional frequency-domain calculation model has good detection ability for small targets, but weak detection ability for large targets. The advantage of our multiscale saliency calculation model is that it can calculate large-scale and small-scale saliency targets at the same time.

The limitation of this work is that it is only concerned with the detection of bottom-up visual saliency. It has not considered top-down influences such as some cues for selecting suitable scales of salience, or some cues for object recognition depending on a given vision task. Future work will focus on a task-dependent attention selection system. It is possible to add top-down influences for developing more intelligent vision systems to accomplish various visual search tasks in engineering applications.

## Data Availability Statement

The original contributions presented in the study are included in the article/supplementary material, further inquiries can be directed to the corresponding author/s.

## Author Contributions

All authors listed have made a substantial, direct, and intellectual contribution to the work and approved it for publication.

## Funding

This work was supported by the National Natural Science Foundation of China (62166048 and 61263048), and by the Applied Basic Research Project of Yunnan Province (2018FB102).

## Conflict of Interest

The authors declare that the research was conducted in the absence of any commercial or financial relationships that could be construed as a potential conflict of interest.

## Publisher's Note

All claims expressed in this article are solely those of the authors and do not necessarily represent those of their affiliated organizations, or those of the publisher, the editors and the reviewers. Any product that may be evaluated in this article, or claim that may be made by its manufacturer, is not guaranteed or endorsed by the publisher.
